# Maternal cobalt concentration and risk of spontaneous preterm birth: the role of fasting blood glucose and lipid profiles

**DOI:** 10.3389/fnut.2024.1336361

**Published:** 2024-02-01

**Authors:** Junhua Huang, Wei Zheng, Aili Wang, Weiling Han, Junxi Chen, Hang An, Lailai Yan, Zhiwen Li, Guanghui Li

**Affiliations:** ^1^Division of Endocrinology and Metabolism, Department of Obstetrics, Beijing Obstetrics and Gynecology Hospital, Capital Medical University. Beijing Maternal and Child Health Care Hospital, Beijing, China; ^2^Department of Obstetrics and Gynecology, Beijing Luhe Hospital, Capital Medical University, Beijing, China; ^3^Institute of Reproductive and Child Health, National Health Commission Key Laboratory of Reproductive Health, Peking University, Beijing, China; ^4^Department of Epidemiology and Biostatistics, School of Public Health, Peking University, Beijing, China; ^5^Department of Laboratorial Science and Technology, School of Public Health, Peking University, Beijing, China

**Keywords:** cobalt, spontaneous preterm birth, fasting blood glucose, lipid profiles, risk

## Abstract

**Introduction:**

Spontaneous preterm birth (SPB) is a significant cause of neonatal mortality, yet its etiology remains unclear. Cobalt, an essential trace element, might be a risk factor for SPB. This study aims to investigate the relationship between maternal serum cobalt concentration and SPB, and to clarify the role of blood lipids and fasting blood glucose (FBG) in this relationship.

**Methods:**

We conducted a nested case-control study within the Beijing Birth Cohort Study. Serum samples were obtained from 222 pregnant women with SPB and 224 controls during the first (7–13 weeks of pregnancy) and third trimesters (32–42 weeks of pregnancy). Serum cobalt concentration was determined using inductively coupled plasma mass spectrometry (ICP-MS). Fasting blood glucose and lipids levels were detected using a fully automated biochemical immunoassay instrument. Logistic regression models and linear regression models were established to explore the association between serum cobalt concentration and the risk of SPB in pregnant women, and to test the mediating effect of fasting blood glucose (FBG) and lipids.

**Results:**

We found that the serum cobalt concentration in mothers with SPB and controls was similar in the first trimester, with values of 0.79 (0.58–1.10) ng/mL and 0.75 (0.51–1.07) ng/mL, respectively. However, in the third trimester, the cobalt concentration increased to 0.88 (0.59–1.14) ng/mL and 0.84 (0.52–1.19) ng/mL, respectively. In the logistic regression model, when considering the third trimester of pregnancy, after adjusting for ethnicity, pre-pregnancy body mass index (BMI), maternal age, education, income, and parity, it was observed that the medium level of cobalt concentration (0.63–1.07 ng/ml) had a negative correlation with the risk of SPB. The odds ratio (OR) was 0.56, with a 95% confidence interval of 0.34–0.90 ng/mL and a *p*-value of 0.02. This suggests that cobalt in this concentration range played a protective role against SPB. Additionally, it was found that FBG in the third trimester of pregnancy had a partial intermediary role, accounting for 9.12% of the association. However, no relationship between cobalt and SPB risk was found in the first trimester.

**Conclusion:**

During the third trimester, intermediate levels of maternal cobalt appear to offer protection against SPB, with FBG playing a partial mediating role. To further clarify the optimal cobalt concentrations during pregnancy for different populations, a multi-center study with a larger sample size is necessary. Additionally, exploring the specific mechanism of FBG’s mediating role could provide valuable insights for improving the prevention of SPB.

## 1 Introduction

Preterm birth refers to delivery before 37 weeks, and it can be categorized into three types: spontaneous delivery with intact membranes, preterm premature rupture of membranes, and iatrogenic preterm birth resulting from medical interventions like labor induction or cesarean section due to maternal-fetal indications. The first two types, which together account for 50 to 70% of preterm births, remain shrouded in mystery regarding their precise causes. Collectively, they are referred to as spontaneous preterm birth (SPB) (1, 2). According to data, the preterm birth rate in the United States in 2019 was 10.2% (3). In China, the rate of preterm birth is also on the rise (4). Each year, approximately 1.1 million newborns die from preterm birth (5) and its related complications, including respiratory distress syndrome (6), apnea (7), metabolic disorders, anemia, and feeding intolerance (8). Even infants who survive may suffer from long-term sequelae such as growth retardation, chronic lung disease, cerebral palsy, and more, posing a significant economic burden on families and society (9). Therefore, reducing the rate of preterm birth, especially the rate of SPB, has become an urgent medical issue that must be addressed.

In recent years, numerous studies have found that the blood concentration of trace elements such as manganese, zinc, selenium, etc. is closely related to the risk of preterm births (10–12). Cobalt, which is well-known as the metal element of vitamin B_12_, exists primarily in cells as divalent and trivalent cation (13). Improper cobalt concentrations can be harmful to one’s health. The Ma’anshan birth cohort (MABC) study examined serum samples from approximately 3,000 individuals and discovered that lower maternal and umbilical cord serum cobalt concentrations were associated with an increased risk of preterm birth (14). Nevertheless, the association between cobalt concentration and the risk of SPB has yet to be reported.

Maternal fasting blood glucose (FBG) and lipid levels also appear to be linked to SPB. Some studies have found that women with SPB exhibit lower total cholesterol (TC), and lower low-density lipoprotein (LDL) levels, along with higher glucose levels (15). This is further compounded by an increased risk of developing type 2 diabetes and cardiovascular disease later in life for these mothers (16, 17). However, the precise mechanisms underlying this association remain unclear, and the conclusions of current studies are inconsistent.

Environmental metallic elements, including cobalt, play a role in lipids and glucose homeostasis. New evidence has shown that environmental cobalt exposure is associated with an increase risk of dyslipidemia (18), as well as impaired fasting blood glucose (19). However, some studies have proposed the opposite viewpoint, indicating that cobalt has a beneficial effect on blood lipids and glucose. This is supported by a negative dose–response relationship with LDL, a positive dose–response relationship with high-density lipoprotein (HDL) (20), and a negative association with FBG (21). The effects of cobalt exposure on blood lipids and glucose are still not fully understood, and the evidence regarding the association between cobalt and glucose and lipid metabolism remains limited.

These lines of evidence have led us to hypothesize that cobalt may contribute to abnormal glucose and lipid metabolism, which, in turn, may jointly contribute to SPB. To investigate this further, we conducted a nested case–control study to examine the relationship between maternal cobalt concentrations and the risk of SPB, as well as to assess whether FBG and blood lipids mediate this association.

## 2 Methods

### 2.1 Study design and participants

**Figure 1:**
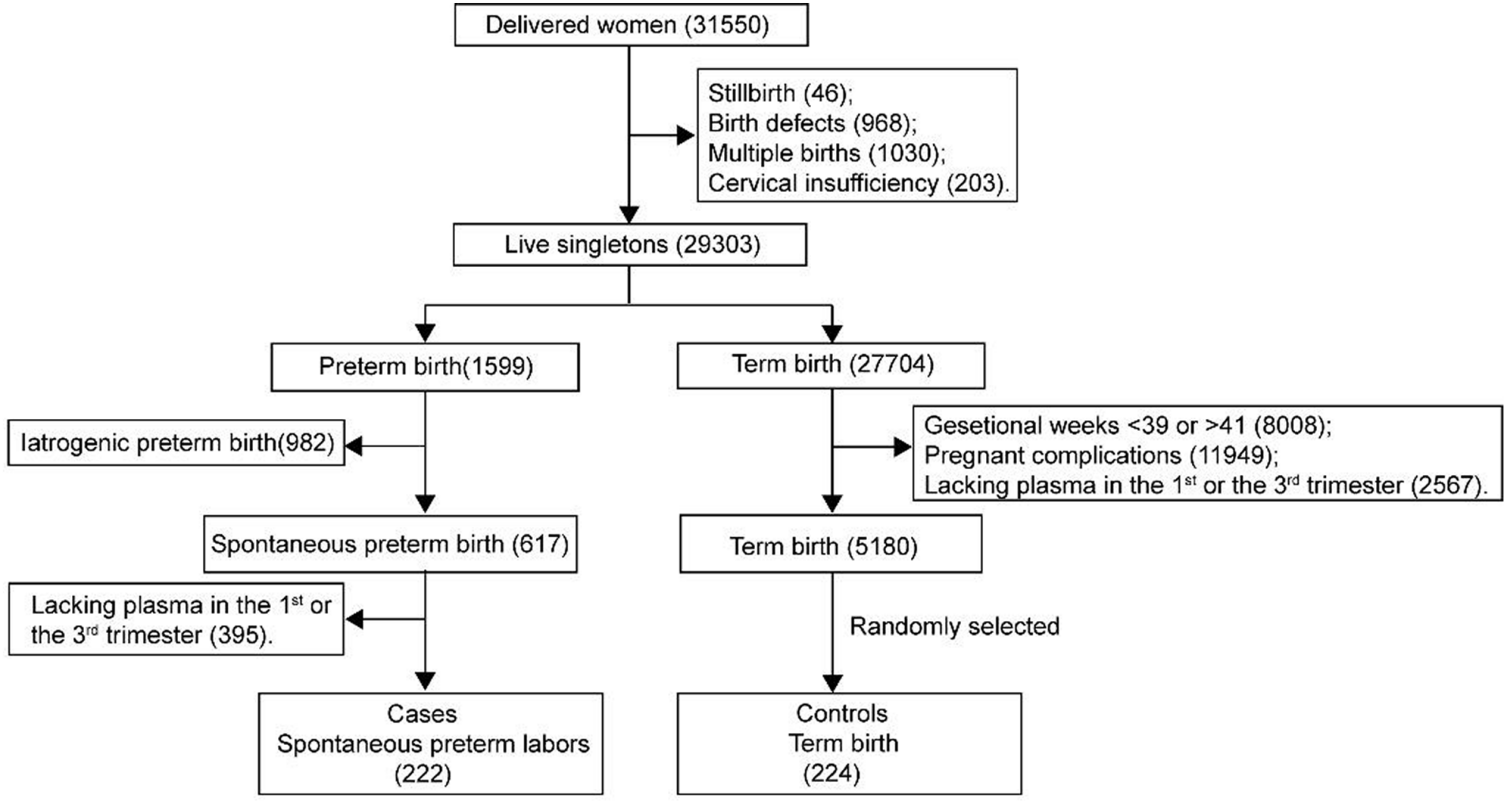
Flow chart of cohort selection of cases and controls.

This study was a nested case–control investigation. Our participants were recruited from the Beijing Birth Cohort Study (BBCS; Number: ChiCTR2200058395), which included 29,303 live births, including 1,599 cases of preterm birth and 617 cases of SPB. Women were eligible for enrollment if they were between 18 and 44 years old and had their first prenatal examination before 14 weeks of gestation. Exclusion criteria included twin pregnancies, cervical insufficiency, history of preterm birth, placental abruption, iatrogenic preterm birth due to conditions such as hypertension, diabetes, central placenta previa, abnormal liver function, acute fatty liver, cholestasis, appendicitis, and major diseases affecting the heart, liver, kidneys, brain, or bed-rest patients. After excluding women who lack first-or third-trimester blood samples, 222 cases of SPB were included in the study. Subsequently, we randomly selected 224 controls from the 5,180 subjects who delivered between 39 and 41 weeks after excluding subjects with complications and those who lacked blood samples (Figure 1). Blood samples were collected at 7–13 and 32–42 weeks of gestation to measure serum cobalt concentrations, lipid profiles and FBG. The study protocol was approved by the Ethics Committee of the Beijing Obstetrics and Gynecology Hospital, Capital Medical University (Number: 2018-KY-009-01). All participants provided written informed consent.

### 2.2 Laboratory analyses

Fasting venous blood (2.0 mL) was collected from the subjects and kept at room temperature for 30 min (The anticoagulant is EDTA). After centrifugation at 4,000 × *g* for 10 min at 4°C, the blood samples were stored in a-80°C freezer for subsequent analysis.

The maternal fasting blood lipid profiles, including TC, triglyceride (TG), HDL, and LDL, were evaluated using an automatic biochemical immunoassay system (Architect ci8200; Abbott Laboratories, Chicago, IL, United States). Additionally, FBG levels were determined by the glucose oxidase method with a DxC800 automatic biochemical analyzer (Beckman Coulter Company, United States).

Inductively coupled plasma mass spectrometry (ICP-MS, ELAN DRC II, PerkinElmer, United States) was utilized to measure the serum cobalt concentration. To ensure accurate results, ClinChek^®^ – Plasma Control, Level II from Germany was used as a quality control standard. The reference value for cobalt in the assay was 9.22 (7.38–11.10) ng/mL. The 0.1 mL of serum was mixed with 0.1 mL of internal standard indium (In, 4 ng/mL) and 1.8 mL of 1% HNO_3_. The samples were then shaken to ensure thorough mixing and were analyzed by the Central Laboratory of Biological Elements at the Peking University Health Science Center. The protocol used in this study was accredited by the Chinese Metrology Accreditation System. The limit of detection (LOD) for cobalt was 0.002 ng/mL, and all samples’ concentrations exceeded this detection limit.

### 2.3 Covariates

In the regression models, the covariates included maternal age (<35, ≥35 years old), pre-pregnancy body mass index (BMI; kg/m^2^; categorized into <18.5, 18.5–25, and ≥ 25), parity (0, ≥1), ethnicity (Han, other ethnicities), education (below Bachelor’s Degree, Bachelor’s Degree, Master’s Degree or above), and income (RMB/month, <10,000, 10,000–20,000, ≥20,000).

### 2.4 Sample size estimation

To the best of our knowledge, there are currently no official guidelines for normal reference values of cobalt in the blood of pregnant women. For the term birth group, we assumed a high-level cobalt exposure rate of approximately 50%. Assuming a risk odds ratio of 2, a significance level of the bilateral test was α = 0.05, β = 0.10, and a 1:1 case–control ratio, the sample size for the SPB group was calculated to be at least 179 cases using the PASS.11 software. Ultimately, we collected 222 cases and 224 controls to assess serum cobalt levels, as well as glucose and lipids levels, during the first and third trimesters of pregnancy.

### 2.5 Statistical analyses

We described numerical variables with normal distributions using mean ± standard deviations, while skewed distributions were described using median (P25–P75). Categorical variables were represented by number (percentage). Given the small proportion of ethnic groups other than Han, we combined these groups (including Manchu, Hui, Korean, Mongol, Yao, and Dong) into a single category for statistical analysis. To compare differences in normal distributed measurement data between two groups, we utilized Student’s *t*-test. For unordered categorical variables and ordinal categorical variables, we employed the Chi-square test and Mann–Whitney U test, respectively. To assess associations between cobalt exposure, blood lipid profiles, FBG and SPB risk, we conducted an unconditional logistic regression analysis, calculating the odds ratio (OR) with a 95% confidence interval (CI), both unadjusted and adjusted for potential confounders. Additionally, we used a linear regression model to evaluate the relationship between cobalt and blood lipid profiles as well as FBG. Different regression equations were established to test for mediating effect. All statistical analyses were performed using SPSS (version 26), and a two-tailed *p*-value <0.05 was considered statistically significant.

## 3 Results

### 3.1 Population characteristics

At baseline, significant differences were observed in maternal pre-pregnancy BMI (*p* = 0.01) and parity (*p* = 0.01) between the SPB and control groups. There was no significant difference in maternal age (*p* = 0.09), education level (*p* = 0.36), income level (*p* = 0.08), and ethnicity (*p* = 0.56) between the two groups. The SPB group delivered at 35.58 ± 0.65 gestational weeks, while the control group delivered at 39.68 ± 0.74 gestational weeks (*p* < 0.01). The infants in the SPB group had a mean length of 47.72 ± 1.96 cm and a mean weight of 2709.96 ± 338.92 g. The control infants had a mean length of 50.27 ± 0.88 cm and a mean weight of 3429.73 ± 296.47 g (Table 1).

**Table 1:** Baseline information.

Variables	SPB (*n* = 222)	Control (*n* = 224)	*p* value
Maternal variables			
Age (years)			0.09
<35	161 (72.52%)	178 (79.46%)	
≥35	61 (27.48%)	46 (20.54)	
Ethnicity			0.56
Han	205 (92.34%)	210 (93.75%)	
Other ethnicities	17 (7.66%)	14 (6.25%)	
Education			0.36
Below bachelor’s degree	59 (26.58%)	42 (18.75%)	
Bachelor’s degree	110 (49.55%)	133 (59.38%)	
Master’s degree or above	53 (23.87%)	49 (21.87%)	
Income (RMB/month)			0.08
<10,000	57 (25.68%)	45 (20.09%)	
10,000–20,000	86 (38.74%)	83 (37.05%)	
≥20,000	79 (35.58%)	96 (42.86%)	
Delivery week, weeks	35.58 ± 0.65	39.68 ± 0.74	<0.01^*^
Pre-pregnancy BMI (kg/m^2^)			0.01*
<18.50	25 (11.26%)	27 (12.05%)	
18.50–25	139 (62.61%)	166 (74.11%)	
≥25	58 (26.13%)	31 (13.84%)	
Parity			0.01*
0 pt	144 (64.86%)	170 (75.89%)	
≥1	78 (35.14%)	54 (24.11%)	
Infant variables			
Infant weighs, g	2709.96 ± 338.92	3429.73 ± 296.47	<0.01^*^
Infant length, cm	47.72 ± 1.96	50.27 ± 0.88	<0.01^*^
Infant gender			0.09
Boy	127 (57.21%)	110 (49.11%)	
Girl	95 (42.79%)	114 (50.89%)	

BMI, body mass index; SPB, spontaneous preterm birth.*p < 0.05.

### 3.2 Serum cobalt concentration, fasting blood glucose, and blood lipid in the case and control group

By comparing cobalt concentration, FBG, and lipids between the two groups during the first and third trimesters, we observed higher levels of TC (4.36 ± 0.71 vs. 4.10 ± 0.64 mmol/L, *p* < 0.01), TG [1.04 (0.79–1.35) vs. 0.87 (0.72–1.12) mmol/L, *p* < 0.01], LDL (2.34 ± 0.60 vs. 2.11 ± 0.54 mmol/L, *p* < 0.01) and FBG (4.68 ± 0.41 vs. 4.60 ± 0.33 mmol/L, *p* = 0.03) in the SPB group compared to the control group during the first trimester. Similarly, in the third trimester, the SPB group had higher levels of TC (6.62 ± 1.18 vs. 6.35 ± 0.95 mmol/L, *p* < 0.01), TG [2.94 (2.49–3.91) vs. 2.61 (2.20–3.27) mmol/L, *p* < 0.01] and FBG (4.37 ± 0.53 vs. 4.24 ± 0.35 mmol/L, *p* < 0.01) compared to the control group. However, cobalt concentrations did not differ significantly between the two groups (Table 2).

**Table 2:** Comparison of cobalt concentration, fasting blood glucose, and lipids between case group and control group.

Biomarkers	SPB (*n* = 222)	Control (*n* = 224)	*p* value
1st trimester			
Cobalt, median (P25–P75), ng/mL	0.79 (0.58–1.10)	0.75 (0.51–1.07)	0.48
Cobalt categories			0.56
<0.60	61 (27.48%)	73 (32.59%)	
0.60–0.97	89 (40.09%)	77 (34.38%)	
≥0.97	72 (32.43%)	74 (33.04%)	
Total cholesterol, mean ± SD, mmol/L	4.36 ± 0.71	4.10 ± 0.64	<0.01*
Triglyceride, median (P25–P75), mmol/L	1.04 (0.79–1.35)	0.87 (0.72–1.12)	<0.01*
High-density lipoprotein, mean ± SD, mmol/L	1.56 ± 0.30	1.58 ± 0.28	0.64
Low-density lipoprotein, mean ± SD, mmol/L	2.34 ± 0.60	2.11 ± 0.54	<0.01*
Fasting blood glucose, mean ± SD, mmol/L	4.68 ± 0.41	4.60 ± 0.33	0.03*
3rd trimester			
Cobalt, median (P25–P75), ng/mL	0.88 (0.59–1.14)	0.84 (0.52–1.19)	0.55
Cobalt categories			0.32
<0.63	57 (25.68%)	78 (34.98%)	
0.63–1.07	95 (42.79%)	71 (31.84%)	
≥1.07	70 (31.53%)	74 (33.18%)	
Total cholesterol, mean ± SD, mmol/L	6.62 ± 1.18	6.35 ± 0.95	<0.01*
Triglyceride, median (P25–P75), mmol/L	2.94 (2.49–3.91)	2.61 (2.20–3.27)	<0.01*
High-density lipoprotein, mean ± SD, mmol/L	1.83 ± 0.32	1.87 ± 0.34	0.22
Low-density lipoprotein, mean ± SD, mmol/L	3.62 ± 1.00	3.45 ± 0.87	0.08
Fasting blood glucose, mean ± SD, mmol/L	4.37 ± 0.53	4.24 ± 0.35	<0.01*

SPB, spontaneous preterm birth; SD, standard deviation.*p < 0.05.

### 3.3 Association between cobalt concentrations and the risk of spontaneous preterm birth

During the third trimester, cobalt concentration in the second tertile (0.63–1.07 ng/mL) was inversely associated with the risk of SPB compared to the first tertile (<0.63 ng/mL), with odds ratios (ORs) of 0.55 (0.35, 0.87), 0.57 (0.35, 0.90), and 0.66 (0.41, 0.93), respectively, after adjusting for various confounding factors (*p* < 0.05). No significant association was observed between cobalt concentration in the first trimester of pregnancy and the risk of SPB (Table 3).

**Table 3:** Logistic regression analysis of maternal cobalt concentration and spontaneous preterm birth.

Cobalt (ng/mL)	SPB delivery week median (IQR)	Odds ratio (95%CI)					
		Model 1^a^	*p* value	Model 2^b^	*p* value	Model 3^c^	*p* value
1st trimester							
Continuous		1.02 (0.70, 1.48)	0.94	0.99 (0.67, 1.45)	0.95	0.97 (0.66, 1.43)	0.88
Categories							
<0.60	36 (35, 36)	1	–	1	–	1	–
0.60–0.97	36 (35, 36)	0.71 (0.45, 1.13)	0.15	0.68 (0.42, 1.08)	0.10	0.62 (0.38, 1.00)	0.05
≥0.97	36 (35, 36)	0.86 (0.57, 1.37)	0.53	0.82 (0.51, 1.32)	0.41	0.78 (0.49, 1.26)	0.31
3rd trimester							
Continuous		0.86 (0.63, 1.18)	0.36	0.85 (0.62, 1.17)	0.32	0.86 (0.62, 1.19)	0.36
Categories							
<0.63	36 (35, 36)	1	–	1	–	1	–
0.63–1.07	36 (35, 36)	0.55 (0.35, 0.87)	0.01*	0.57 (0.35,0.90)	0.02*	0.66 (0.41, 0.93)	0.03*
≥1.07	36 (35, 36)	0.77 (0.48, 1.24)	0.29	0.78 (0.48,1.27)	0.32	0.88 (0.54, 1.41)	0.58

SPB, spontaneous preterm birth; CI, confidence interval; IQR, interquartile range.

a Unconditional logistic regression with no adjustments.

b Unconditional logistic regression with adjustments for pre-pregnancy BMI and parity.

c Unconditional logistic regression with adjustments for ethnicity, pre-pregnancy BMI, maternal age, education, income, and parity.

*p < 0.05.

### 3.4 Involvement of fasting blood glucose and lipids in the association between maternal cobalt concentration and the risk of spontaneous preterm birth

To assess the mediating effect of FBG and lipids, we first evaluated linear regression models for cobalt concentration and FBG and lipids. The results are presented in Table 4. In the first trimester, when the cobalt concentration was <0.60 ng/mL, it was negatively correlated with FBG (β = −0.14; 95%CI, −0.19 to-0.04, *p* < 0.01). Conversely, when cobalt concentration was between 0.60 ng/mL and 0.97 ng/mL, it was positively related to FBG (β = 0.10; 95%CI, 0.01 to 0.15, *p* = 0.04). In the third trimester, when cobalt concentration was <0.63 ng/mL, it was negatively associated with FBG (β = −0.10; 95%CI, −0.19 to-0.01, *p* = 0.04). No significant relationship was observed between cobalt concentration and blood lipids (Table 4).

**Table 4:** Linear regression models of fasting blood glucose and lipids according to maternal cobalt concentration.

Cobalt (ng/mL)	Fasting blood glucose		Total cholesterol		Triglyceride		Low-density lipoprotein		High-density lipoprotein	
	β (95%CI)	*p* value	β (95%CI)	*p* value	β (95%CI)	*p* value	β (95%CI)	*p* value	β (95%CI)	*p* value
1st trimester										
Continuous	0.04 (−0.04, 1.00)	0.38	−0.03 (−0.17, 0.09)	0.55	−0.01 (−0.08, 0.08)	0.94	−0.03 (−0.14, 0.08)	0.58	−0.05 (−0.08, 0.03)	0.31
<0.60	−0.14 (−0.19, −0.04)	<0.01*	−0.01 (−0.15, 0.13)	0.88	0.01 (−0.07, 0.10)	0.78	0.01 (−0.11, 0.13)	0.84	0.01 (−0.06, 0.06)	0.93
0.60–0.97	0.10 (0.01, 0.15)	0.04*	0.03 (−0.09, 0.18)	0.52	0.01 (−0.08, 0.08)	1.00	0.02 (−0.09, 0.14)	0.68	0.01 (−0.05, 0.06)	0.93
≥0.97	0.03 (−0.05, 0.10)	0.48	−0.03 (−0.17, 0.10)	0.60	−0.01 (−0.09, 0.07)	0.79	−0.03 (−0.15, 0.08)	0.54	−0.01 (−0.06, 0.05)	0.86
3rd trimester										
Continuous	0.10 (0.01, 0.14)	<0.01*	−0.01 (−0.17, 0.17)	1.00	0.01 (−0.17, 0.17)	0.99	−0.01 (−0.06, 0.05)	0.79	0.02 (−0.12, 0.17)	0.71
<0.63	−0.10 (−0.19, −0.01)	0.04*	0.05 (−0.11, 0.33)	0.33	−0.02 (−0.26, 0.18)	0.71	0.04 (−0.04, 0.10)	0.40	0.04 (−0.10, 0.28)	0.35
0.63–1.07	0.06 (−0.04, 0.14)	0.25	0.01 (−0.21, 0.21)	1.00	−0.02 (−0.25, 0.18)	0.74	−0.01 (−0.07, 0.06)	0.80	0.01 (−0.18, 0.18)	0.99
≥1.07	0.04 (−0.05, 0.13)	0.42	−0.05 (−0.32, 0.11)	0.33	0.03 (−0.14, 0.30)	0.48	−0.03 (−0.09, 0.05)	0.50	−0.04 (−0.27, 0.10)	0.36

CI, confidence interval. All models were adjusted for parity and pre-pregnancy BMI.*p < 0.05.

We then utilized logistic regression models to assess the impact of FBG and lipids on the SPB. In the first, FBG (Table 5, OR = 0.58; 95%CI, 0.35 to 0.96, *p* = 0.03), TC (Table 5, OR = 0.57; 95%CI, 0.43 to 0.76, *p* < 0.01), TG (Table 5, OR = 0.36; 95%CI, 0.22 to 0.58, *p* < 0.01), and LDL (Table 5, OR = 0.48; 95%CI, 0.34 to 0.68, *p* < 0.01) were all negatively associated with the risk of SPB. The significance of TC, TG, and LDL persisted after adjustments for different factors. In the third trimester, FBG (Table 5, OR = 0.51; 95%CI, 0.33 to 0.80, *p* < 0.01), TC (Table 5, OR = 0.79; 95%CI, 0.66 to 0.94, *p* = 0.01) and TG (Table 5, OR = 0.60; 95%CI, 0.49 to 0.74, *p* < 0.01) were also negatively associated with SPB, and these findings remained unchanged after adjustments for ethnicity, pre-pregnancy BMI, maternal age, education, income, and parity.

**Table 5:** Logistic regression analysis of spontaneous preterm birth according to fasting blood glucose and lipids.

Biomarkers	Odds ratio (95%CI)					
	Model 1^a^	*p* value	Model 2^b^	*p* value	Model 3^c^	*p* value
1st trimester						
Fasting blood glucose	0.58 (0.35, 0.96)	0.03*	0.68 (0.40, 1.16)	0.16	0.69 (0.40, 1.18)	0.18
Total cholesterol	0.57 (0.43, 0.76)	<0.01*	0.58 (0.43, 0.78)	<0.01*	0.57 (0.43, 0.78)	<0.01*
Triglyceride	0.36 (0.22, 0.58)	<0.01*	0.43 (0.26, 0.71)	<0.01*	0.43 (0.26, 0.72)	<0.01*
Low-density lipoprotein	0.48 (0.34, 0.68)	<0.01*	0.51 (0.36, 0.73)	<0.01*	0.50 (0.35, 0.72)	<0.01*
High-density lipoprotein	1.17 (0.61, 2.24)	0.64	0.94 (0.47,1.86)	0.85	0.96 (0.48, 1.93)	0.92
3rd trimester						
Fasting blood glucose	0.51 (0.33, 0.80)	<0.01*	0.59 (0.37, 0.94)	0.03*	0.57 (0.35, 0.92)	0.02*
Total cholesterol	0.79 (0.66, 0.94)	0.01*	0.70 (0.57, 0.85)	<0.01*	0.70 (0.570.85)	<0.01*
Triglyceride	0.60 (0.49, 0.74)	<0.01*	0.61 (0.49, 0.75)	<0.01*	0.61 (0.49, 0.76)	<0.01*
Low-density lipoprotein	0.83 (0.68, 1.02)	0.08	0.71 (0.57, 0.89)	<0.01*	0.72 (0.58, 0.90)	<0.01*
High-density lipoprotein	1.43 (0.80, 2.55)	0.22	1.34 (0.75, 2.42)	0.33	1.33 (0.73, 2.43)	0.36

CI, confidence interval.

a Unconditional logistic regression with no adjustments.

b Unconditional logistic regression with adjustments for pre-pregnancy BMI and parity.

c Unconditional logistic regression with adjustments for ethnicity, pre-pregnancy BMI, maternal age, education, income, and parity.

*p < 0.05.

**Figure 2:**
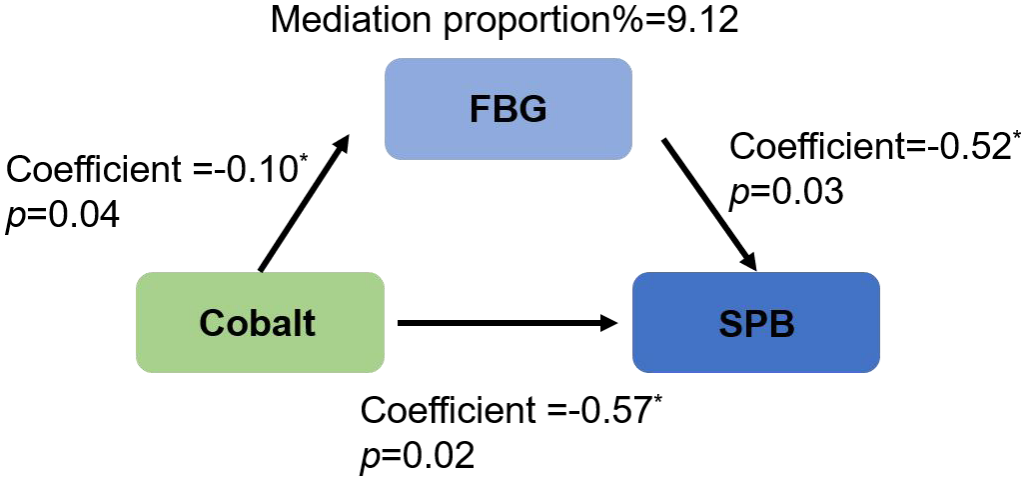
Partial mediation of fasting blood glucose in the association between maternal cobalt concentration and spontaneous preterm birth in the third trimester. SPB, spontaneous preterm birth; FBG, fasting blood glucose.

The initial logistic regression analysis suggested a negative correlation between cobalt and SPB (Table 3, β = −0.57; OR = 0.57; 95%CI, 0.35 to 0.90; *p* = 0.02). Subsequently, in the linear regression analysis (Table 4), cobalt concentration was inversely associated with FBG (β = −0.10; 95%CI, −0.19 to-0.01, *p* = 0.04). In the second logistic regression analysis (Table 5), FBG had a significant impact on SPB (β = −0.52, OR = 0.59, 95%CI, 0.37 to 0.94, *p* = 0.03). When cobalt concentration was between 0.63 to 1.07 ng/mL in the third trimester, both cobalt and FBG had significant effects on SPB (Table 6, β = −0.57; OR = 0.57; 95%CI, 0.35 to 0.93, *p* = 0.02), indicating that FBG partially mediated this relationship. According to the results of the coefficient test, the mediating effect was calculated as follows: |(−0.10)* (−0.52)/(−0.57)|*100% = 9.12% (Figure 2).

**Table 6:** Logistic regression analysis of spontaneous preterm birth according to cobalt concentration and fasting blood glucose in the third trimester.

Cobalt (ng/mL)	Odds ratio (95%CI)							
	Model 1^a^	*p* value	Model 2^b^	*p* value	Model 3^c^	*p* value	Model 4^d^	*p* value
Continuous	0.86 (0.63, 1.18)	0.36	0.91 (0.67, 1.25)	0.57	0.89 (0.65, 1.23)	0.48	0.91 (0.65, 1.26)	0.55
<0.63	1	–	1	–	1	–	1	–
0.63–1.07	0.55 (0.35, 0.87)	0.01*	0.55 (0.35,0.89)	0.02*	0.57 (0.35, 0.93)	0.02*	0.56 (0.34, 0.90)	0.02*
≥1.07	0.77 (0.48, 1.24)	0.29	0.85 (0.52, 1.38)	0.51	0.84 (0.51, 1.38)	0.49	0.95 (0.58, 1.55)	0.83

CI, confidence interval.

a Logistic regression analysis of spontaneous preterm birth according to cobalt concentration with no adjustments.

b Logistic regression analysis of spontaneous preterm birth according to cobalt concentration and fasting blood glucose with no adjustments.

c Logistic regression analysis of spontaneous preterm birth according to cobalt concentration and fasting blood glucose with adjustments for pre-pregnancy BMI and parity.

d Logistic regression analysis of spontaneous preterm birth according to cobalt concentration and fasting blood glucose with adjustments for ethnicity, pre-pregnancy BMI, maternal age, education, income, and parity.

*p < 0.05.

## 4 Discussion

There have been limited prior studies exploring the link between cobalt and the risk of SPB, and to date, no reports have delved into the role of intermediary variables in this relationship. Our study revealed that, when compared to the lowest tertile of cobalt concentrations in the third trimester of pregnancy, cobalt concentrations ranging from 0.63 to 1.07 ng/mL may play a protective role in SPB. Furthermore, FBG acts as a partial mediator in this context.

Our finding that lower cobalt concentrations were protective against SPB aligns with previous research highlighting the adverse health effects associated with high cobalt exposure. Inhalation of dust containing high levels of cobalt can lead to inflammation and fibrosis in the bronchi and lungs (22). Skin contact with high amounts of cobalt can trigger allergic reactions (23, 24). Additionally, elevated urinary cobalt concentration has been positively associated with obesity, insulin resistance, increased TG, and decreased HDL (25). However, no prior studies have examined the association between cobalt and the risk of SPB. The Ma’anshan birth cohort study in China reported a link between maternal and umbilical cord serum cobalt levels and the risk of preterm birth. Their findings indicated that lower levels of these cobalt concentrations were associated with an elevated risk of preterm birth. It is noteworthy that their study collected maternal blood samples during the first and second trimesters of pregnancy, while our study focused on the first and third trimesters. This difference in timing could potentially explain the discrepancies in our findings. In the Ma’anshan birth cohort data (14), the median serum cobalt concentration among mothers who experienced preterm birth (*n* = 132) in the first and second trimesters was 157.03 ng/L with an interquartile range of 122.10–203.80 ng/L and 335.60 ng/L with a range of 220.96–485.66 ng/L, respectively. These values were significantly lower than those observed in our study among mothers who were SPB, which were 790 (580–1,100) ng/L and 880 (590–1,140) ng/L in the first and third trimesters, respectively. It is possible that the higher cobalt levels in their cohort align with the lower levels in our cohort, indicating that the results of our two studies might not be as contradictory as initially thought. In another Korean study, no significant differences were observed in cobalt concentrations between healthy pregnant women and those who experienced preterm births, regardless of whether concentrations were above or below 730 ng/L (26). The concentration of cobalt in serum varies greatly among different populations, influenced by factors such as diet, occupation, and living environment. Therefore, it is challenging to pinpoint the specific reasons for these variations (13).

In the general population, including the participants of this study, the primary source of cobalt intake is through food. Notably, Brazil nuts, caw liver (27), and herbs (28) are rich in cobalt. Other dietary items such as fish, green leafy vegetables, coffee, and grains also provide significant amounts of cobalt (13). However, we did not investigate the dietary habits of the study population, thus preventing an assessment of cobalt intake through this route of external exposure. This was a limitation of our study. Other exposure routes to cobalt include occupational exposure, which is primarily observed in the hard metal industry; environmental exposure, which includes cobalt distributed in air, soil, and water, as well as exposure from electronic devices, jewelry, cosmetics, and leather goods; and medical exposure, including cobalt-containing pharmaceutical preparations and metal joint prostheses (29). The pregnant women in our study did not have occupational exposure to miners or heavy metal factory workers, and therefore were not exposed to cobalt through their occupations. Environmental pollution primarily resulted from cobalt mines (30, 31), and there was no related pollution near the residences of the study participants. Therefore, this form of cobalt exposure can be ruled out in our study. When it comes to electronic products, lithium-ion batteries in portable electronics and electric cars have been the primary users of cobalt. These devices, including mobile phones, televisions, and computers, appear to have minimal toxic effects on the human body (32), with limited research available on their impact. Cobalt is also present in cosmetics (such as lipstick, eyeshadow, and soap) (33, 34), jewelry (35), and leather products (36), primarily entering the body through skin contact. Pregnant women in our study may have been exposed to cobalt through the use of these products during pregnancy. However, we did not collect data on the specific cosmetics, jewelry, and leather products used by the participants, preventing us from assessing cobalt exposure from these sources. Future studies could explore exposure to cobalt and other trace elements from these perspectives. Cobalt is also commonly utilized in medical orthopedic implants (such as hip joints) and medical imaging (cobalt isotopes) (37). Cobalt ferrite nanoparticles have become essential magnetic nanomaterials in biomedical applications and are widely used in biosensors, drug delivery, magnetic separation, and purification (38). Our study population did not have exposure to these medical applications of cobalt, and therefore this external source of cobalt exposure can be excluded.

We discovered a negative correlation between serum cobalt and SPB, with FBG playing a partial mediating role. Notably, cobalt concentration was also negatively associated with FBG. While the FBG of the SPB group exceeded that of the control group, it remained below the diagnostic cut-off value of 5.1 mmol/L for gestational diabetes mellitus (39). Therefore, there was no blood glucose abnormality among our study participants. It is possible that the higher FBG in the SPB group was solely due to cobalt exposure, though the precise mechanism remains unknown. Previous research has also identified an inverse association between plasma cobalt and FBG in men (21), while another study found a similar inverse relationship between urinary cobalt and high FBG in metal-exposed workers (40). In contrast to the aforementioned findings, other studies have reported a positive correlation between urinary cobalt and the increased prevalence of impaired fasting glucose (19). Furthermore, a higher cobalt content has been observed in whole blood samples of the diabetic population (41). Coincidentally, cobalt is significantly associated with a reduction in fasting plasma glucose levels in elderly individuals and women without hyperlipidemia (42).

This study has several limitations. Firstly, due to the nature of the observational study design, we cannot fully eliminate the influence of residual confounding factors. However, this is a prospective case–control study, and all cases and controls are derived from the same cohort population. The baseline characteristics of the study population are relatively homogeneous, which helps to minimize confounding factors by ensuring a similar genetic and exposure background. Secondly, some variables such as maternal alcohol consumption, passive smoking, diet, nutritional supplement intake, and lifestyle during pregnancy were not included in the initial design. We plan to incorporate surveys on these factors in future studies. Finally, our study population consisted of residents living in urban areas of Beijing, so the conclusions drawn from this study may not be applicable to other regions or populations. Nevertheless, the serum cobalt concentration we measured can reflect changes in the body’s circulation and metabolism following exposure to various factors over a prolonged period of time. This concentration can also serve as a proxy comprehensive cobalt exposure to some extent.

Our study had several noteworthy strengths. Firstly, we conducted a prospective nested case–control study, minimizing potential selection and recall biases. Our sample size was adequate, particularly in the SPB group, providing valuable insights into the role of cobalt in pregnant women with SPB. Measuring cobalt concentrations prior to delivery offers a preventive perspective on SPB and enables us to assess the potential causal relationship between maternal cobalt levels and SPB. The analysis of mediating variables between cobalt and SPB offers new perspectives for future research into the mechanisms underlying cobalt’s association with preterm birth. Finally, we employed a repeated measures design to illuminate the dynamic changes in maternal serum cobalt concentrations throughout pregnancy. By focusing solely on SPB cases, we effectively distinguished them from iatrogenic preterm birth, thereby eliminating any confounding effects associated with iatrogenic preterm birth.

## 5 Conclusion

Our research has revealed that during the third trimester of pregnancy, intermediate levels of cobalt may serve a protective function in relation to SPB. Furthermore, FBG plays a partial mediating role. It is important to note that the cobalt concentrations observed in our study population were higher compared to other studies, and our findings cannot be generalized to other populations. Therefore, future studies with larger sample sizes and multi-center designs are necessary to clarify the optimal cobalt concentration range for different pregnant women groups throughout pregnancy. Additionally, further investigation into the specific mechanism of FBG’s mediating role is essential to provide valuable insights for effective prevention strategies against SPB.

## Data availability statement

The original contributions presented in the study are included in the article/supplementary material, further inquiries can be directed to the corresponding author/s.

## Ethics statement

The study protocol was approved by the Ethics Committee of the Beijing Obstetrics and Gynecology Hospital, Capital Medical University (Number: 2018-KY-009-01). The studies were conducted in accordance with the local legislation and institutional requirements. The participants provided their written informed consent to participate in this study.

## Author contributions

JH: Conceptualization, Formal analysis, Investigation, Writing – original draft, Writing – review & editing. WZ: Investigation, Supervision, Writing – review & editing. AW: Data curation, Investigation, Writing – review & editing. WH: Data curation, Investigation, Writing – review & editing. JC: Methodology, Software, Writing – review & editing. HA: Methodology, Software, Writing – review & editing. LY: Methodology, Validation, Writing – review & editing. ZL: Funding acquisition, Project administration, Resources, Writing – review & editing. GL: Funding acquisition, Project administration, Resources, Supervision, Visualization, Writing – review & editing.
