# Supplementary material for: Maternal cobalt concentration and risk of spontaneous preterm birth: the role of fasting blood glucose and lipid profiles
Source: Front Nutr. 2024 Feb 1;11:1336361. doi: 10.3389/fnut.2024.1336361 (PMC10867207; doi:10.3389/fnut.2024.1336361)
Supplement: Supplementary file 1 [file Table_1.docx]

**Maternal Cobalt Concentration and Risk of Spontaneous Preterm Birth: The Role of Fasting Blood Glucose and Lipid Profiles**

Junhua Huang^1^, Wei Zheng^1^, Aili Wang^1,2^, Weiling Han^1^, Junxi Chen^3,4^, Hang An^3,4^, Lailai Yan^5^, Zhiwen Li^3,4*^, Guanghui Li^1*^

^1^ Division of Endocrinology and Metabolism, Department of Obstetrics, Beijing Obstetrics and Gynecology Hospital, Capital Medical University. Beijing Maternal and Child Health Care Hospital, Beijing, China.

^2^ Department of Obstetrics and gynecology, Beijing Luhe Hospital, Capital Medical University, Beijing, China.

^3^ Institute of Reproductive and Child Health, National Health Commission Key Laboratory of Reproductive Health, Peking University, Beijing, China.

^4^ Department of Epidemiology and Biostatistics, School of Public Health, Peking University, Beijing, China.

^5^ Department of Laboratorial Science and Technology, School of Public Health, Peking University, Beijing, China.

Supplementary Table 1. Logistic regression for stratified analyses according to pre-pregnancy BMI

| Pre-pregnancy BMI (kg/m2) | Cobalt (ng/mL) | Numbers (n) | | Odds ratio (95%CI) | | | | | |
| --- | --- | --- | --- | --- | --- | --- | --- | --- | --- |
|  |  | SPB | Control | Model 1^a^ | *P* value | Model 2^b^ | *P* value | Model 3^c^ | *P* value |
| 1^st^ trimester |  |  |  |  |  |  |  |  |  |
| <18.50 | Continuous | 25 | 27 | 1.02 (0.70, 1.48) | 0.94 | 0.99 (0.68, 1.46) | 0.97 | 0.98 (0.66, 1.44) | 0.90 |
|  | <0.36 | 2 | 9 | 1 | - | 1 | - | 1 | - |
|  | 0.36-0.99 | 13 | 9 | 0.71 (0.45, 1.12) | 0.14 | 0.65 (0.41, 1.04) | 0.07 | 0.62 (0.39, 1.00) | 0.05 |
|  | ≥0.99 | 10 | 9 | 0.86 (0.54, 1.36) | 0.51 | 0.82 (0.51, 1.30) | 0.39 | 0.78 (0.48, 1.25) | 0.29 |
| 18.50-25 | Continuous | 152 | 176 | 1.11 (0.73, 1.70) | 0.62 | 1.10 (0.72,1.69) | 0.67 | 1.07 (0.70, 1.65） | 0.75 |
|  | <0.36 | 25 | 26 | 1 | - | 1 | - | 1 | - |
|  | 0.36-0.99 | 81 | 97 | 0.75 (0.44, 1.26) | 0.28 | 0.75 (0.44, 1.29) | 0.30 | 0.72 (0.42， 1.25) | 0.24 |
|  | ≥0.99 | 46 | 53 | 0.88 (0.51, 1.52) | 0.64 | 0.84 (0.48, 1.47) | 0.55 | 0.80 (0.46，1.42) | 0.80 |
| ≥25 | Continuous | 45 | 21 | 1.15 (0.34, 3.89) | 0.83 | 1.06 (0.31, 3.59) | 0.93 | 0.92 (0.24, 3.58) | 0.91 |
|  | <0.36 | 10 | 4 | 1 | - | 1 | - | 1 | - |
|  | 0.36-0.99 | 21 | 9 | 0.55 (0.10, 3.22) | 0.51 | 0.61 (0.10, 3.88) | 0.60 | 0.39 (0.05, 2.87) | 0.36 |
|  | ≥0.99 | 14 | 8 | 1.23 (0.40, 3.85) | 0.72 | 1.23 (0.39, 3.91) | 0.50 | 1.07 (0.32, 3.65) | 0.91 |
| 3^rd^ trimester |  |  |  |  |  |  |  |  |  |
| <18.50 | Continuous | 25 | 27 | 0.91 (0.67, 1.25) | 0.57 | 0.89 (0.65, 1.23) | 0.48 | 0.91 (0.65, 1.26) | 0.55 |
|  | <0.55 | 7 | 9 | 1 | - | 1 | - | 1 | - |
|  | 0.55-0.92 | 9 | 9 | 0.64 (0.40, 1.03) | 0.06 | 0.66 (0.41, 1.07) | 0.10 | 0.66 (0.41, 1.08) | 0.10 |
|  | ≥0.92 | 9 | 9 | 0.94 (0.59, 1.51) | 0.80 | 0.93 (0.58, 1.50) | 0.77 | 0.95 (0.58, 1.55) | 0.83 |
| 18.50-25 | Continuous | 152 | 175 | 0.77 (0.53, 1.12) | 0.17 | 0.73 (0.50, 1.06) | 0.10 | 0.74 (0.50, 1.09) | 0.13 |
|  | <0.55 | 33 | 48 | 1 | - | 1 | - | 1 | - |
|  | 0.55-0.92 | 49 | 50 | 0.66 (0.38, 1.15) | 0.14 | 0.67 (0.38, 1.19) | 0.17 | 0.69 (0.38, 1.22) | 0.20 |
|  | ≥0.92 | 70 | 77 | 0.88 (0.51, 1.52) | 0.65 | 0.86 (0.49, 1.49) | 0.59 | 0.88 (0.50, 1.54) | 0.65 |
| ≥25 | Continuous | 45 | 21 | 3.07 (1.11, 8.48) | 0.03* | 3.03 (1.10, 8.36) | 0.03* | 4.19 (1.22, 14.38) | 0.02* |
|  | <0.55 | 10 | 5 | 1 | - | 1 | - | 1 | - |
|  | 0.55-0.92 | 14 | 7 | 0.48 (0.12, 1.96) | 0.31 | 0.41 (0.10, 1.71) | 0.22 | 0.26 (0.05, 1.35) | 0.11 |
|  | ≥0.92 | 21 | 9 | 0.20 (0.05, 0.84) | 0.03* | 1.93 (0.47. 7.97) | 0.36 | 4.16 (0.70, 24.7) | 0.12 |

SPB, spontaneous preterm birth; CI, confidence interval; BMI, body mass index.

^a^Unconditional logistic regression with adjustments for fasting blood glucose.

^b^ Unconditional logistic regression with adjustments for fasting blood glucose and parity.

^c^Unconditional logistic regression with adjustments for fasting blood glucose, ethnicity, maternal age, education, income, and parity.

*p<0.05.

Supplementary Table 2. Logistic regression for stratified analyses according to parity

| Parity | Cobalt concentration | Numbers (n) | | Odds ratio (95%CI) | | | | | |
| --- | --- | --- | --- | --- | --- | --- | --- | --- | --- |
|  |  | SPB | Control | Model 1^a^ | *P* value | Model 2^b^ | *P* value | Model 3^c^ | *P* value |
| 1st trimester |  |  |  |  |  |  |  |  |  |
| 0 | Continuous | 144 | 170 | 1.00 (0.63, 1.62) | 0.97 | 1.00 (0.62, 1.60) | 0.98 | 0.98 (0.61, 1.58) | 0.94 |
|  | <0.36 | 28 | 27 | 1 | - | 1 | - | 1 | - |
|  | 0.36-0.99 | 71 | 87 | 0.81 (0.47, 1.39) | 0.44 | 0.73 (0.42, 1.28) | 0.27 | 0.70 (0.40, 1.23) | 0.21 |
|  | ≥0.99 | 45 | 56 | 1.03 (0.60, 1.76) | 0.92 | 1.00 (0.58, 1.71) | 1.00 | 0.96 (0.55, 1.66) | 0.87 |
| ≥1 | Continuous | 78 | 54 | 1.08 (0.57, 2.06) | 0.82 | 0.98 (0.50, 1.89) | 0.94 | 1.03 (0.51, 2.10) | 0.93 |
|  | <0.36 | 9 | 12 | 1 | - | 1 | - | 1 | - |
|  | 0.36-0.99 | 44 | 28 | 0.56 (0.24, 1.31) | 0.18 | 0.50 (0.21, 1.20) | 0.12 | 0.36 (0.14, 0.95) | 0.04* |
|  | ≥0.99 | 25 | 14 | 0.55 (0.22, 1.36) | 0.20 | 0.48 (0.19, 1.25) | 0.13 | 0.423(0.15, 1.24) | 0.12 |
| 3rd trimester |  |  |  |  |  |  |  |  |  |
| 0 | Continuous | 144 | 169 | 0.79 (0.55, 1.14) | 0.21 | 0.79 (0.54, 1.13) | 0.20 | 0.80 (0.55, 1.17) | 0.26 |
|  | <0.55 | 33 | 47 | 1 | - | 1 | - | 1 | - |
|  | 0.55-0.92 | 41 | 48 | 0.74 (0.42, 1.31) | 0.30 | 0.74 (0.42, 1.30) | 0.29 | 0.70 (0.40, 1.25) | 0.23 |
|  | ≥0.92 | 70 | 74 | 0.89 (0.51, 1.56) | 0.68 | 0.90 (0.51, 1.58) | 0.71 | 0.92 (0.52, 1.65) | 0.79 |
| ≥1 | Continuous | 78 | 54 | 1.27 (0.66, 2.44) | 0.48 | 1.42 (0.72, 2.82) | 0.32 | 1.64 (0.78, 3.46) | 0.19 |
|  | <0.55 | 17 | 15 | 1 | - | 1 | - | 1 | - |
|  | 0.55-0.92 | 31 | 18 | 0.46 (0.19, 1.08) | 0.07 | 0.45 (0.19, 1.10) | 0.08 | 0.64 (0.24, 1.72) | 0.38 |
|  | ≥0.92 | 30 | 21 | 1.00 (0.41, 2.43) | 1.00 | 0.47 (0.19, 1.21) | 0.12 | 1.33 (0.49, 3.62) | 0.58 |

SPB, spontaneous preterm birth; CI, confidence interval.

^a^Unconditional logistic regression with adjustments for fasting blood glucose.

^b^ Unconditional logistic regression with adjustments for fasting blood glucose and pre-pregnancy body mass index.

^c^Unconditional logistic regression with adjustments for fasting blood glucose, ethnicity, pre-pregnancy BMI, maternal age, education, and income.

^*p<0.0^
